# The axis of long non-coding RNA MALAT1/miR-1-3p/CXCR4 is dysregulated in patients with diabetic neuropathy

**DOI:** 10.1016/j.heliyon.2022.e09178

**Published:** 2022-03-24

**Authors:** Donya Ashjari, Negin Karamali, Misagh Rajabinejad, Seyedeh Sara Hassani, Leila Afshar Hezarkhani, Daryoush Afshari, Ali Gorgin Karaji, Farhad Salari, Alireza Rezaiemanesh

**Affiliations:** a Student Research Committee, School of Medicine, Kermanshah University of Medical Sciences, Kermanshah, Iran; b Department of Immunology, School of Medicine, Kermanshah University of Medical Sciences, Kermanshah, Iran; c Student Research Committee, School of Medicine, Mazandaran University of Medical Sciences, Sari, Iran; d Department of Immunology, School of Medicine, Mazandaran University of Medical Sciences, Sari, Iran; e Department of Neurology, School of Medicine, Kermanshah University of Medical Sciences, Kermanshah, Iran

**Keywords:** Diabetic neuropathy, MALAT1, miR-1-3p, CXCR4

## Abstract

**Background:**

Diabetic neuropathy (DN) is a prevalent complication of diabetes mellitus characterized by pain and inflammation. Long non-coding RNAs (lncRNAs) have been associated with DN. This study aimed to investigate transcript levels of Metastasis-associated lung adenocarcinoma transcript 1 (MALAT1), microRNA (miR)-1-3p, and C-X-C motif chemokine receptor 4 (CXCR4) in the DN patients and type 2 diabetes mellitus (T2DM) cases without neuropathy.

**Methods:**

Here, 20 cases with DN and 20 T2DM subjects without neuropathy (as the control group) were included. Total RNA was extracted from peripheral blood mononuclear cells (PBMCs) of all participants. The expression levels of targets were evaluated by Real-time-PCR.

**Results:**

Results showed that MALAT1 (Fold change = 2.47, *P* = 0.03) and CXCR4 (Fold change = 1.65, *P* = 0.023) were significantly upregulated, while miR-1-3p was downregulated (Fold change = 0.9, *P* = 0.028) in whole blood samples from DN patients compared to the control group. A significant correlation was found between transcript levels of MALAT1 and CXCR4 (*rho* = 0.84; *P* < 0.0001).

**Conclusions:**

This study suggests a possible involvement of the MALAT1/miR-1-3p/CXCR4 axis in the pathogenesis of DN.

## 1 Introduction

Diabetic neuropathy (DN) is a prevalent and debilitating complication of diabetes mellitus (DM) that is characterized by nerve damage and pain [1]. Among the most prevalent microvascular problems associated with diabetes is DN. The most common manifestations of DN are suppressed sensation, intense pain, and promoted risk of foot ulcers [2]. Hyperglycemia is among the numerous factors associated with a high risk of DN [3].

The chemokine C-X-C motif ligand 12 (CXCL12) and its receptor, C-X-C motif chemokine receptor 4 (CXCR4), are expressed on a wide range of cells that are involved in inflammation and organogenesis [4, 5]. This chemokine is also a neuromodulator in the nervous system and plays an essential role in developing and maintaining pathological pain [6, 7]. One of the major causes of painful diabetic neuropathy (PDN) is DN [8]; pain is one of the most significant symptoms of this disease that severely affects the quality of life in the patients [9]. Numerous studies have shown that CXCR4 and its ligand play a vital role in developing and maintaining pain in PDN patients [10, 11, 12].

Non-coding RNAs (ncRNAs) are a significant group of RNA transcripts without a protein-coding function [13]. ncRNAs include two groups of housekeeping and regulatory. The main classes of regulatory ncRNAs are microRNAs (miRNAs; containing 19–25 nucleotides) and long non-coding RNAs (lncRNAs; containing more than 200 nucleotides) [14]. Both miRNAs and lncRNAs regulate gene translation through different mechanisms [15].

Different studies indicate that miRNAs are involved in the pathogenesis and diagnosis of diabetes. Diabetes is associated with changes in miRNA profiles in the blood that can be used as diagnostic biomarkers to predict the disease as well as associated complications [16]. Metastasis-associated lung adenocarcinoma transcript 1 (MALAT1), located on chromosome 11q13, is a lncRNA that plays an essential role in cell proliferation, differentiation, and migration. Studies showed that MALAT1 is involved in the pathogenesis of diabetes and types of cancers. Additionally, the findings also point out the relation between MALAT1 and miR-1-3p. MALAT1 through acting as miRNA sponges binds to miR-1-3p and prevents its interaction with target mRNAs [17, 18, 19, 20, 21].

Here in this study, we investigated the expression levels of MALAT-1, miR-1-3p, and CXCR4 in the peripheral blood samples and evaluate their relation in DN patients.

## 2 Materials and methods

### 2.1 Participants

This cross-sectional study included type 2 diabetes mellitus (T2DM) patients without any neuropathic complications (n = 20) and DN patients (n = 20). The protocol of the study was endorsed by the ethical committee of Kermanshah University of Medical Sciences (Ethical code: IR.KUMS.REC.1399.576) for use of human participants. Patients were selected randomly from those attending the Farabi clinic and Imam Reza hospital in Kermanshah, Iran. T2DM was determined according to the "Report of the Expert Committee on the Diagnosis and Classification of Diabetes Mellitus" [22, 23]. DN was also confirmed by a neurologist based on Electromyography (EMG), Nerve Conduction Velocity (NCV), and blood tests. The control group was randomly selected from patients with T2DM who had been diagnosed with T2DM for more than 5 years. An internal medicine doctor then confirmed their diabetes with a blood test, and a neurologist performed EMG and NCV tests to rule out neuropathy. Patients were excluded if they had a history of autoimmune or immunodeficiency diseases, taking insulin shots, and other medications except for gabapentin and nortriptyline. Written informed consent was obtained from all participants before sampling. Epidemiological data and demographic indicators (history of diabetes, surgery or hospitalization, drug or alcohol use, smoking status) were collected, and patients were matched for age, sex, Gabapentin, and Nortriptyline use.

### 2.2 Bioinformatics analysis

To predict miRNA targets, we used Targetscan, miRanda, and miRDB databases. Expression of the miRNA target gene was also assessed through the GeneCards databases. LncRNAdb, NONCODE, and StarBase were used to identify specific lncRNA regulating miR-1-3. The DIANA algorithm, a sensitive and specific program for miRNA target prediction, was used to perform the *in silico* computations [24].

### 2.3 RNA preparation

After collecting the peripheral blood samples, in order to standardize for cell count and CBC, before starting to run the samples each time, the cell counter was calibrated. PBMCs were isolated using Ficoll-Hypaque gradient density centrifugation. Then, RNA extraction from PBMCs was performed with NORGEN Biotek Total RNA Purification Kit (Norgen Biotek, Inc., Thorold, ON, Canada) based on the manufacturer's instructions. We used NanoDrop 2000 (ThermoScientific) to evaluate the quality of the obtained RNA, based on an optical density (OD) of 260/280 ratio ≥1.8 (indicating RNA contamination with protein) and the OD of 260/230 ratio ≥2.0 (indicating RNA contamination with organic compounds).

### 2.4 Quantitative Real-time polymerase chain reaction (qRT-PCR)

Complementary DNA (cDNA) synthesis was conducted with miRCURY LNA RT Kit (Qiagen, Inc., Hilden, Germany) for miRNAs and nRT-ROSET Kit (ROJETechnologies Inc., Yazd, Iran) for mRNA detection by a Thermocycler (Bio RAD Thermal Cycler C1000 Touch system). The qRT-PCR was performed using Real-time PCR technique (LighCycler 96® instrument, Roche Applied Science, Penzberg, Germany) exerting the SYBER green Premix (Ampliqon, Denmark) to evaluate the expression of CXCR4 and MALAT1 and the miRCURY LNA miRNA PCR Assays kit (QIAGEN, Germany) for detecting miR-1-3p transcript level. The primers (QIAGEN, Germany) used for quantification of the expression level of the mRNA, miRNA, and lncRNA are summarized in Table 1. To evaluate the specificity of the products, the melting curves were analyzed. As the reference controls for normalization of miR-1-3p expression as well as CXCR4 and MALAT1 expressions, transcript levels of U6 snRNA and 18S rRNA, respectively, were used. Relative changes in the transcriptional levels of the targets were determined by using the relative quantification method and the 2^−ΔΔCt^ formula described by *pfaffl* [25]. qRT-PCR was conducted in duplicate for each sample.

**Table 1:** Primer sequences used in the quantitative Real-time PCR.

Type of gene	Gene name	Primer sequence (5′–3′)
Target gene	MALAT1	F: GACGGAGGTTGAGATGAAGC
		R: ATTCGGGGCTCTGTAGTCCT
	CXCR4	F: ATCTGCCTCACTGACGTTGG
		R: TGGTCTATGTTGGCGTCTGG
Reference gene	18s rRNA	F: GTAACCCGTTGAACCCCATT
		R: CCATCCAATCGGTAGTAGCG
	U6 snRNA	F: CTCGCTTCGGCAGCACA
		R: AACGCTTCACGAATTTGCGT

MALAT1, Metastasis-associated lung adenocarcinoma transcript 1; CXCR4, C-X-C motif chemokine receptor 4.

### 2.5 Statistical analysis

Data analysis and graph designing were performed by GraphPad Prism version 8 (GraphPad, Software, La Jolla, California, USA). Mann Whitney *U* test was used to compare transcript levels between two groups. Spearman’s rho test was used for performing correlation analysis. Data were displayed as the mean ± standard error of the mean; (SEM). A *p*-value < 0.05 was set to be statistically significant.

## 3 Results

### 3.1 Demographic data and laboratory findings

We listed the laboratory findings and baseline characteristics of the participants in Table 2. We measured FBS for all study participants. The cholesterol levels were not significantly different between the two groups, but fasting blood sugar (FBS; *P* < 0.001), Hemoglobin A1c (HbA1c; *P* < 0.05), and duration of diabetes demonstrated a significant increase in DN patients compared to non-DN T2DM patients.

**Table 2:** Characteristics of the participants included in the study.

Variables		Diabetic neuropathy (n = 20)	Type 2 diabetes mellitus (n = 20)	*P* value
Age; year (Mean ± SEM)		58.20 ± 1.44	57.60 ± 1.83	0.66
Sex; male/female		12 (60%)/8 (40%)	6 (30%)/14 (70%)	0.11
Duration of diabetes; year		13.92 ± 0.9	8.35 ± 0.82	<0.0001
Drugs	Gabapentin	+ (20)	- (20)	-
	Nortriptyline	+ (20)	- (20)	-
	Insulin	- (20)	- (20)	-
	FBS	238.95 ± 19.3	155.27 ± 13.29	<0.0001
	HbA1c∗	9.35 ± 0.44 (n = 19)	7.97 ± 0.34 (n = 13)	0.02
	Cholesterol	166.33 ± 11.43	166.60 ± 8.85	ns

SEM, Standard error of mean; FBS, Fasting blood sugar; HbA1c, Hemoglobin A1c.

∗ Data for HbA1c was available for 19 patients with diabetic neuropathy and 13 cases with type 2 diabetes mellitus.

### 3.2 MALAT1 expression level

**Figure 1:**
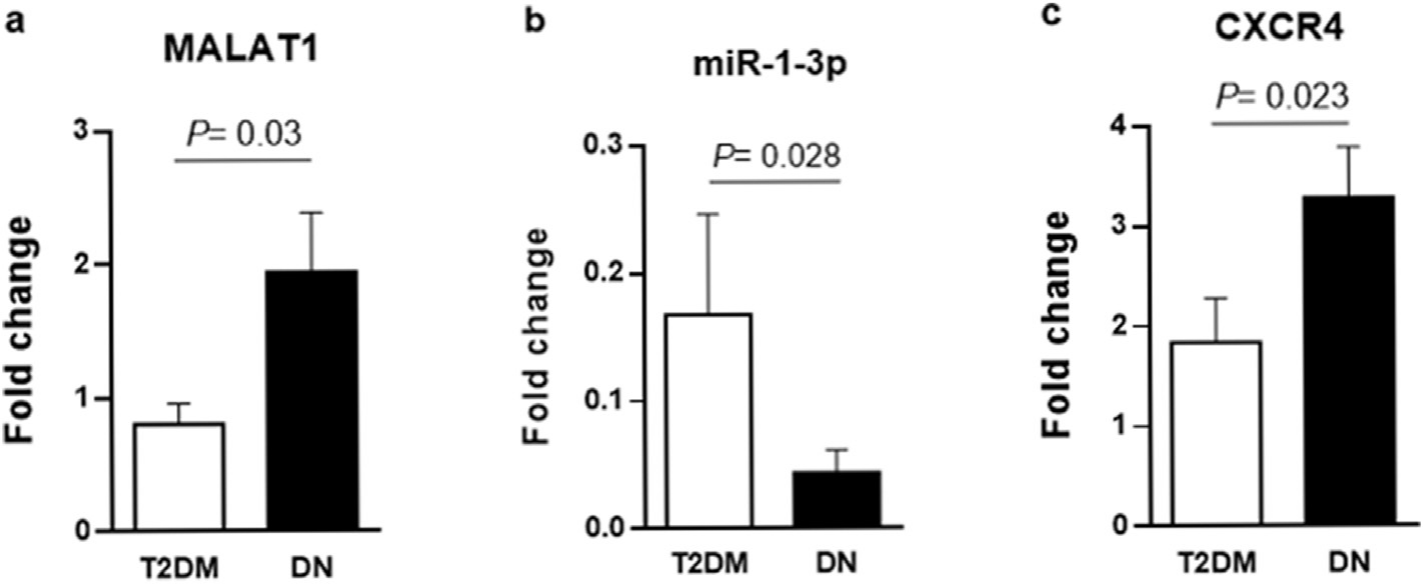
Relative gene expression of MALAT1 (A), miR-1-3p (B), and CXCR4 (C). Real-time PCR was used to detect the transcriptional level of targets in the peripheral blood samples obtained from diabetic neuropathy (DN) and type 2 diabetes mellitus (T2DM) subjects as the control group. Mann Whitney U test was used to compare transcript levels of the targets between two groups.

The results displayed a significantly increased expression of MALAT1 in PBMCs from DN cases compared to the T2DM group (Fold change = 2.47, *P* = 0.03; Figure 1a).

### 3.3 miR-1-3p expression level

A significant decrease in miR-1-3p expression level was observed in PBMCs from DN patients relative to the T2DM group (Fold change = 0.9, *P* = 0.028; Figure 1b).

### 3.4 CXCR4 expression level

It was observed that CXCR4 expression was significantly upregulated in PBMCs from the DN group in comparison to the T2DM group (Fold change = 1.65, *P* = 0.023; Figure 1c).

### 3.5 Correlation analysis

**Figure 2:**
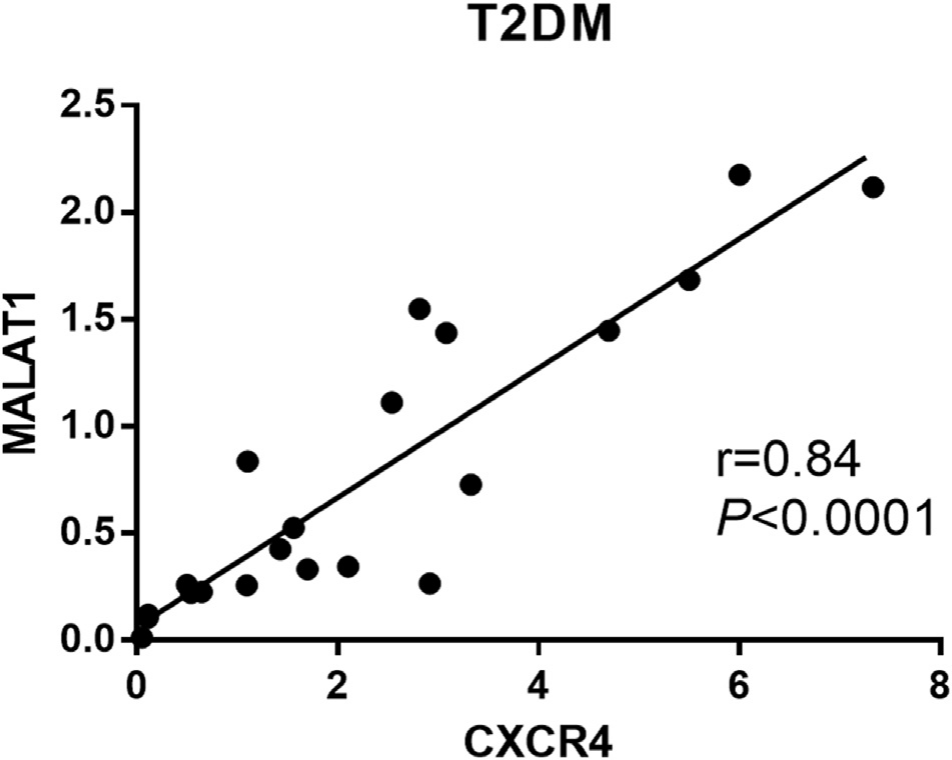
Correlation dot plot and related regression line for significant correlations between MALAT1 and CXCR4 in type 2 diabetes mellitus (T2DM) subjects.

The results of the correlation analysis between the transcript levels of MALAT1, miR-1-3p, and CXCR4, and laboratory parameters (FBS, cholesterol, HbA1c, and duration of diabetes) in the study groups is shown in Table 3. The expression levels of MALAT1 and CXCR4 in T2DM patients had significant correlations (*rho* = 0.84; *P* < 0.0001; Figure 2).

**Table 3:** Correlations between expression levels of MALAT1, miR-1-3p, CXCR4, and clinical characteristics in the study groups.

	MALAT1		miR-1-3p		CXCR4	
	DN	T2DM	DN	T2DM	DN	T2DM
FBS						
*rho* (*P* value)	0.01 (0.96)	-0.24 (0.38)	0.07 (0.79)	0.14 (0.62)	(0.42) 0.08	(-0.17) 0.55
HbA1c	0.12 (0.63)	-0.26 (0.40)	0.32 (0.23)	-0.47 (0.19)	-0.17 (0.51)	-0.50 (0.13)
Cholesterol	-0.03 (0.89)	-0.01 (0.94)	-0.39 (0.16)	0.08 (0.81)	-0.04 (0.87)	0.05 (0.86)
Duration of diabetes	-0.15 (0.49)	-0.04 (0.84)	-0.21 (0.40)	-0.10 (0.69)	0.30 (0.19)	-0.02 (0.92)
MALAT1	-	-	0.13 (0.59)	0.02 (0.91)	-0.12 (0.62)	**0.84 (<0.0001)**
miR-1-3p	0.13 (0.59)	0.02 (0.91)	-	-	0.41 (0.10)	0.13 (0.64)

MALAT1, Metastasis-associated lung adenocarcinoma transcript 1; miR, microRNA; CXCR4, C-X-C motif chemokine receptor 4; FBS, Fasting blood sugar; HbA1c, Hemoglobin A1c; DN, diabetic neuropathy; T2DM type 2 diabetes mellitus. Data in bold show statistically significant (*p* ​< 0.05) comparisons.

## 4 Discussion

To date, the mechanisms underlying the etiopathogenesis of neuropathy in diabetic patients have not yet been fully understood. Previous studies have shown the pathogenic role of inflammation in the development of DN [26]. In a 2011 study, Yamakawa *et al.* indicated that TNF-α plays an important role in diabetic neuropathy and that using infliximab as a monoclonal anti-TNF-α-antibody improves this disease [27]. Purwata *et al.* investigated the role of TNF-α and nitric oxide (NO) in the pathogenesis of painful diabetic neuropathy and found that in the patients with diabetic neuropathy, higher levels of TNF-α and higher inducible NO synthase (iNOS) and TNF-α immunoreactivity of macrophages increased the risk of pain and painful diabetic neuropathy [28]. Various studies have shown that CXCR4 plays a crucial role in causing PDN; in these patients, CXCR4 was shown to be increased in the spinal and dorsal root ganglia (DRG) neurons that elevated calcium influx, reduced the pain threshold, and was also involved in inflammation [7, 29, 30, 31]. A study by da Silva Junior *et al.* reported that AMD3100, CXCR4 antagonist, reduced interleukin (IL)-6 and the calcium influx in diabetic synaptosomes and inhibited the Stromal cell-derived factor 1 (SDF1)-induced hypersensitivity in a model of Streptozocin (STZ)-induced PDN in Wistar rats through CXCR4-antagonist properties [32]. In the present study, we compared the CXCR4 expression level in PBMCs of DN patients to that of T2DM patients. The results demonstrated a significant upregulation in CXCR4 expression in the DN group. In addition to the inflammatory and diagnostic role of CXCR4 in DN, it may be used as a biomarker to monitor the disease progression toward PDN.

Numerous studies have demonstrated that miRNAs are associated with different types of human diseases [33]. In a study by McClelland *et al.*, an essential role of miRNAs in the progression of diabetes mellitus and its complications was established [34]. Guo *et al.* study revealed that miR-1-3p expression was decreased in DRG from STZ-induced diabetic rats while the expression of its target gene, Mgat4a, was upregulated [35]. A study reported that miR-1-3p expression was decreased in T2DM cases compared with controls. In addition, a correlation was detected between miR-1-3p with HbA1c levels in patients [36]. Our results also showed a decrease in miR-1-3p expression in DN cases compared with the T2DM patients. Nonetheless, the transcript level of miR-1-3p was not correlated with clinical and laboratory findings (FBS, HbA1c, cholesterol, and duration of diabetes) (Table 3).

The association of MALAT1 with inflammatory factors has been shown in various studies [37, 38, 39]. Different studies have shown that MALAT1 expression is increased in diabetes-related complications, including diabetic cardiomyopathy, diabetic kidney disease, diabetic retinopathy, and gestational diabetes [40, 41, 42, 43, 44]. Hu *et al.* demonstrated that MALAT1 expression was increased in the kidney cortex from mice with STZ-induced DN. They also found that β-catenin plays a role in the transcription of MALAT1 [45]. Controversially, a study by Tello-Flores *et al.* reported that the expression of MALAT1 in the serum or serum exosome of T2DM patients was reduced. Also, the transcript level of MALAT1 was correlated with cholesterol in T2DM patients [46]. Even though we found upregulation of MALAT1 in the PBMCs from DN cases compared with T2DM cases, the transcript level of MALAT1 was not correlated with clinical and laboratory data in the patients (Table 3).

Studies on various malignancies have shown that MALAT1 is associated with miR-1-3p, and inhibition of MALAT1 leads to increased levels of miR-1-3p and, hence, decreased expression of its target gene [17, 20]. MALAT1 was shown to plays a role in the proliferation, migration, and invasion of human hilar cholangiocarcinoma (HCCA) cells. By silencing the MALAT1 gene, it was also found that CXCR4 expression was downregulated and miR-204 expression was upregulated [47]. The results of our study showed that the MALAT1 expression was upregulated in DN patients compared with the control group. There was also a significant correlation between MALAT1 and CXCR4 in DN patients. However, increased MALAT-1 transcript level did not correlate with downregulated miR-1-3p level. In this regard, to confirm the association between MALAT1 and CXCR4 in DN, it is necessary to evaluate the expression of CXCR4 by silencing MALAT1 in future studies.

## 5 Conclusion

In summary, this study indicated that MALAT1 and CXCR4 were upregulated while miR-1-3p was downregulated in DN patients compared with the T2DM subjects; it was also found that expression levels of MALAT1 were correlated with CXCR4 in DN patients. One of the limitations of the present study is the lack of patients' neural samples as well as experimental models. In addition, the expression of CXCR4 varies in different immune cells (highest in monocytes and B cells, and lower in T cells and NK cells), and therefore the results obtained in this study may not be generalizable to all peripheral blood leukocytes involved in the inflammatory process in DN. Considering the possible involvement of MALAT1/miR-1-3p/CXCR4 axis in the pathogenesis of DN raised by our pilot study, further studies are suggested in the future exerting more samples alongside patient classification based on the quality of hyperglycemia control in order to precisely clarify the role of this axis in the etiopathogenesis of DN.

## Declarations

### Author contribution statement

Donya Ashjari: Performed the experiments; Wrote the paper.

Negin Karamali: Analyzed and interpreted the data; Wrote the paper.

Misagh Rajabinejad: Conceived and designed the experiments; Analyzed and interpreted the data.

Seyedeh Sara Hassani: Performed the experiments.

Leila Afshar Hezarkhani, Daryoush Afshari: Contributed reagents, materials, analysis tools or data.

Ali Gorgin Karaji, Farhad Salari: Analyzed and interpreted the data.

Alireza Rezaiemanesh: Conceived and designed the experiments; Wrote the paper.

### Funding statement

This research has been supported by grants from Kermanshah University of Medical Sciences (KUMS); Grant No. 990581.

### Data availability statement

Data included in article/supp. material/referenced in article.

### Declaration of interests statement

The authors declare no conflict of interest.

### Additional information

No additional information is available for this paper.
